# Our Data, Our Truths, Our Voice: Reclaiming First Nations Data Sovereignty in Manitoba

**DOI:** 10.23889/ijpds.v9i5.2731

**Published:** 2024-09-18

**Authors:** Stephanie Sinclair, Jillian Waruk, Taylor Morrisieau, Carla Cochrane, Ashley Saulog

**Affiliations:** 1 First Nation Health and Social Secretariat of Manitoba

## Background

Indigenous data sovereignty applies to the collection, ownership, and application of data about Indigenous peoples, lands, and resources.

## Objective

To support and expand Manitoba First Nations (FNs) data sovereignty by strengthening capacity and policies related to the ownership, repatriation, and governance of FNs health data.

## Approach

In March of 2022, the Chiefs of the 63 First Nations in Manitoba adopted a resolution to mandate FNHSSM to implement the First Nations Data Governance Strategy. FNHSSM Data Sovereignty Team has conducted extensive engagement with FNs (including youth, Knowledge Keepers, urban members, 2SLGBTQIA+ members, health directors, and more) to identify current priorities for data collection, research, policy, and training. Basic analysis was done to determine the frequency with which each theme and sub-theme were discussed.

## Results

Based on the engagement sessions themes that refer to traditional FNs cultural practices and knowledge represented a major priority for all groups, including the integration of strength-based indicators of wellbeing. Overall, FNs have reinforced the urgent need to exert ownership over community health data under the principles of data sovereignty. The health directors top three themes include: (1) data repatriation, (2) governance, ethics, and privacy and, (3) mental health, substance use, violence and poverty.

## Conclusions

Through engagement, we have identified data needs (including access, training, policies), data topics (health priorities, traditional practices), and data uses (advocacy, repatriation, research) that are unique to FNs in Manitoba. Our team is working in collaboration with FNs to gain greater control over their data, enhance their data analysis capabilities, and ensure the protection of privacy and culture.

## Implications

Operationalizing data sovereignty will support evidence-based decision making, research capacity, governance, and ultimately, self-determination.

